# Three Millennia of Nanocrystals

**DOI:** 10.1021/acsnano.1c11159

**Published:** 2022-03-24

**Authors:** Federico Montanarella, Maksym V. Kovalenko

**Affiliations:** † Laboratory of Inorganic Chemistry, Department of Chemistry and Applied Biosciences, ETH Zürich, Vladimir-Prelog-Weg 1, CH-8093 Zürich, Switzerland; ‡ Laboratory for Thin Films and Photovoltaics, Empa−Swiss Federal Laboratories for Materials Science and Technology, Überlandstrasse 129, CH-8600 Dübendorf, Switzerland

**Keywords:** Quantum dots, nanocrystals, history, colloids, luster ceramic, stained glass, gold, lead halide perovskites

## Abstract

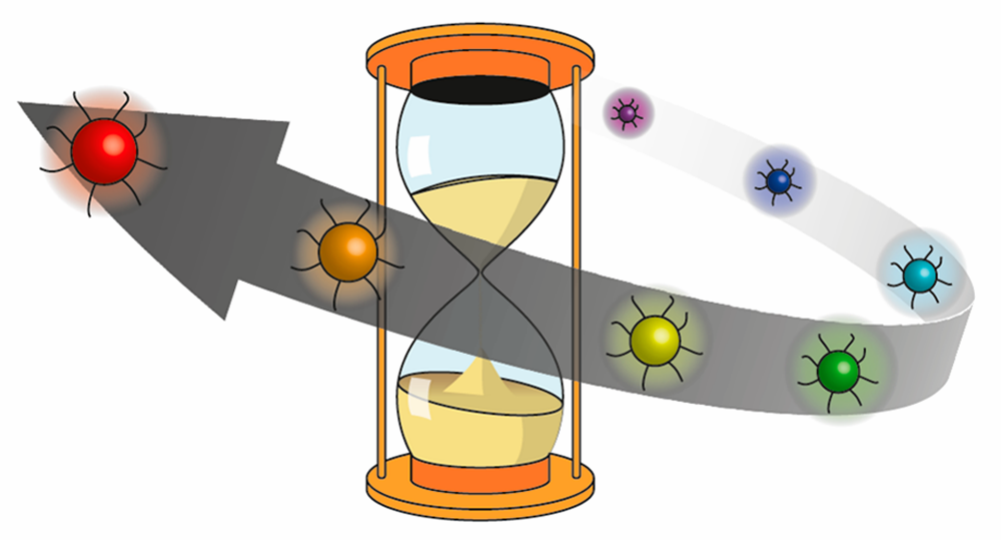

The broad deployment of nanotechnology and nanomaterials in modern society is increasing day by day to the point that some have seen in this process the transition from the Silicon Age to a new Nano Age. Nanocrystals—a distinct class of nanomaterials—are forecast to play a pivotal role in the next generation of devices such as liquid crystal displays, light-emitting diodes, lasers, and luminescent solar concentrators. However, it is not to be forgotten that this cutting-edge technology is rooted in empirical knowledge and craftsmanship developed over the millennia. This review aims to span the major applications in which nanocrystals were consistently employed by our forebears. Through an analysis of these examples, we show that the modern-age discoveries stem from multimillennial experience passed on from our proto-chemist ancestors to us.

The birth of nanotechnology—that is, the use of materials with nanoscale dimensions and/or whose properties rely on their structural organization at the nanoscale—is usually associated with two events: the speech by Richard Feynman in 1959 at Caltech (“There is Plenty of Room at the Bottom”, December 29, American Physical Society meeting),^1^ and the speech by Norio Taniguchi in 1974 (“On the basic concept of Nanotechnology”) at the International Conference on Production Engineering in Tokyo.^2^ Since these two events, the use of the word *nano* (denoting a factor of 10^–9^) emerged in both scientific and nonscientific literature. In the 1990s, thanks to actions encouraging and coordinating research in the nanodomain, such as the National Nanotechnology Initiative in the United States,^3^ an association between the terms “nanotechnology” and “future” was promoted in the collective image.^4−7^ Currently, the use of nanotechnologies and nanomaterials is seen in a growing number of commercial applications. For instance, nanosized metal–oxide–semiconductor field-effect transistors have been implemented in the last generations of computers and smartphones.^8−10^

Semiconductor and metal nanocrystals (NCs) have evolved into much-appraised material’s building blocks in nanoscience and nanotechnology.^11,12^ In recent decades, giant leaps have been made in the development, optimization, and commercialization of nanocrystals for diverse purposes;^12−15^ for example, sunscreens contain ZnO and TiO_2_ nanoparticles as absorbers of the ultraviolet fraction of sunlight,^16−18^ while semiconductor nanocrystals can be found in some of the most modern liquid-crystal displays (LCDs) to achieve a better color gamut.^19^ This drive is motivated by the awareness that their macroscopic properties depend on the nanocrystal dimensions and size. For instance, the much higher surface-to-volume ratio compared to their bulk counterparts produces unmatched catalytic properties in nanoparticles,^20−26^ as was shown, for example, by Paul Sabatier when he used ultrafine nickel particles to perform catalytic hydrogenation (which gained him the Nobel Prize in 1912).^27^ Furthermore, nanotechnology and, in particular, nanocrystals are forecast to play a pivotal role in the next generation of devices in electronics,^28,29^ medicine,^30−34^ photonics,^35−38^ and catalysis^22^ as well as in less conventional fields^39−41^ such as textiles.^42^ The use of nanomaterials is shaping our society to the point that some have recognized in it the birth of a new age (i.e., the Nano Age), distinguishing it from the current “Silicon Age”.^43,44^

However, as is often the case in science, the use of nanotechnology can be traced back far earlier in time. In fact, history presents a plethora of situations in which nanotechnology was used in purely empirical and unwitting, though effective, approaches.^45−48^ During the fifth millennium BC the inhabitants of Cyprus would bleach wool and fleece with nanoporous clay,^49,50^ while Corsica has a long-standing tradition of (nano)asbestos-enriched pottery as a way to enhance the mechanical properties of clay.^51^ The Mesoamerican civilization of the Maya^52^ developed two weather-resistant pigments based on the incorporation of natural dyes into nanostructured clay.^53−57^ At the same time, the Christian crusade warriors could probably not conceive that the superior properties of the Moorish Damascus steel were due to embedded carbon nanotubes,^58,59^ although this type of nanotechnology was already known and used almost a millennium earlier in India.^60^ Concerning nanocrystals, despite the common notion that they are a relatively recent technology, they have also been used, though (again) purely empirically, for at least three millennia. We will also show that their modern (re)discovery stems from a long-standing traditional use. As we will show in this review, the modern field of nanocrystals relies on an ancient, deep, and factual know-how that can be traced back historically as far as the ancient world. We will first discuss how nanocrystals were widely used in the manufacturing of glass. Notably, the case of gold will be discussed more extensively as a prototypical example of the development of nanocrystals through the centuries. Related to glass and gold, we will then examine the use of nanocrystals in the coloration of ceramics. An analysis of the use of nanocrystals for cosmetics will follow. We will then proceed to the brief history of semiconductor nanocrystals and, in particular, how it evolved from the glass-making industry.

## Nanocrystals in Glass

**Figure 1:**
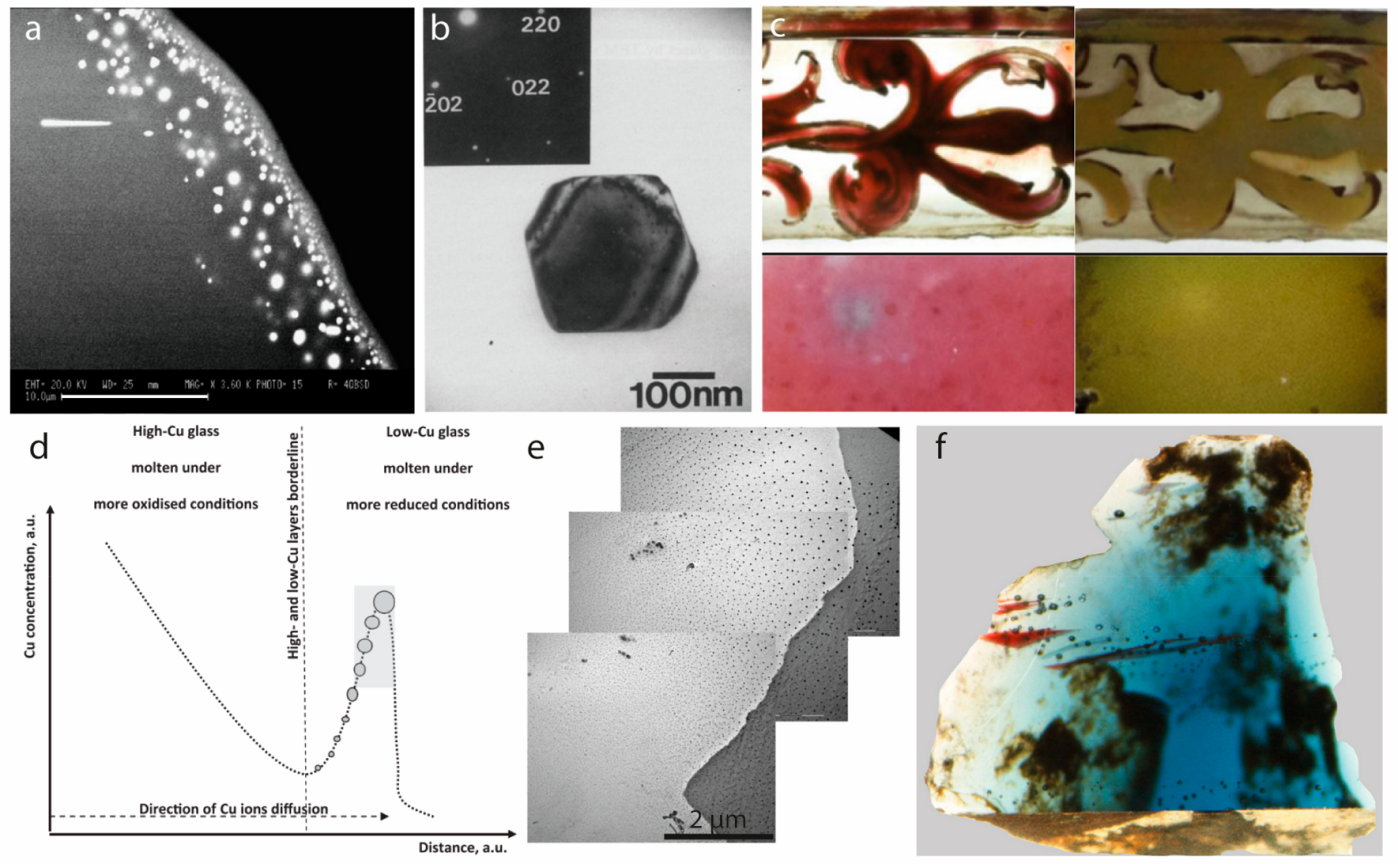
Examples of the use of nanocrystals in glass throughout history. (a) Scanning electron microscopy (SEM) image of Cu nanocrystals dispersed in a Bronze Age colored glass from Frattesina di Rovigo (Italy); reprinted with permission from ref (61). Copyright (2004), Elsevier. (b) Transmission electron microscopy (TEM) image of a metallic Cu crystallite in a Gallo-Roman glass (4th century AD); reprinted with permission from ref (66). Copyright (1991) Chapman & Hall, with permission of Springer Nature. (c) Stained glass piece from the cathedral of Avila (16th century), Spain, observed through optical microscopy in transmitted light (left) and reflected light (right); reprinted with permission from ref (67). Copyright (2013) Springer Science Business Media Dordrecht. (d) Scheme representing the distribution, form, and concentration of Cu in red glass. The darker shade zone represents the development of red color; reprinted with permission from ref (68). Copyright (2013) Elsevier. (e) Composite TEM image of a Cu red glass from Burgos, Spain, 13th century. The black dots, whose size increases from bottom left to top right, are Cu nanocrystals; reprinted with permission from ref (68). Copyright (2013) Elsevier. (f) Cobalt blue glass from York Minister (England), 15th century, presenting red *striae* from precipitation of Cu nanocrystals; reprinted with permission from ref (68). Copyright (2013) Elsevier.

The development of nanocrystals is intimately related to the field of glass manufacturing: already in the ancient world, glass was, in fact, colored through the addition of metals to the melt. Some of the earliest archeological evidence suggests that, already during the Bronze Age, in Italy, our ancestors enriched silica pastes with chromophores (e.g., Cu, Co, Fe, and Mo) in order to color glass. In particular, red glass was obtained through the formation of metallic copper nanocrystals from the reduction of copper oxides under a reducing atmosphere.^61^ These metallic copper nanocrystals, typically located at the surface of the glass (Figure 1a), rendered blue colored glasses with a thin red colored layer originating either from the excitation of localized surface plasmon modes in the nanoparticles or by scattering from the nanoparticles themselves.^62−64^ This evidence shows that, even though our ancestors were not able to produce glass in a standardized, scalable manner, they mastered this art well enough to manufacture colored glass with controlled macroscopic properties, using an empirical technique very similar to that used to produce luster ceramics and modern colored glass more than two millennia later.^65^

The same technique of coloration developed in the Bronze Age and relying on metallic Cu nanocrystals (Figure 1b) was maintained throughout the centuries, when it was, for example, used to produce red enamels and mosaic tesserae in both Celtic and Roman Gaul (current-day France).^66,69^ However, the technique of coloring with Cu nanocrystals was finally exploited in all its potentiality with the production of the huge stained glass windows of the Romanic and, especially, Gothic cathedrals (e.g., Sainte-Chapelle in Paris) during the Middle Age. In the whole Christendom, glassmakers refined and improved the manufacturing of colored glass through the centuries, extending it to incorporate the use of silver to obtain a yellow/amber coloration. The use of silver was most probably adopted from the Middle East, where it was already known from the eighth century AD in the production of luster ceramics. The first report in the Christian world seems to appear in the 13th century in the “El Lapidario” of king Alfonso X, where a stone called *Ecce* (most probably pyrargyrite, Ag_3_SbS_3_) is suggested to be ground up, mixed with honey, and spread over glass in order to dye it in yellow/gold.^67,70^ Recent studies unearthed the mechanism of production of these types of glass: a silver-based slurry paste was applied to soda-lime glass (rich in Na and K compounds), which was then annealed (300–600 °C).^71,72^ In a process that is very similar to that used for luster pottery,^73^ the Ag^+^ ions would diffuse into the glass, while Na^+^ and K^+^ ions would migrate toward the surface; the thermoreducing agents in the glass (e.g., As^3+^, Sb^3+^, Sn^2+^) would then reduce the silver ions to Ag° nanocrystals with a diameter of 5–10 nm.^71,74^ The glassmakers were able to control the resulting color of the glass through the presence of additives in the glass: the presence of Cu ions, for example, would slow down Ag^+^ diffusion, thus producing larger nanocrystals and hence obtaining more vivid colors.^75^ The same technique was used to obtain dichroic glasses (Figure 1c): a longer growth of Ag nanocrystals would typically result in nanocrystal populations with different sizes in the same glass and therefore with optical properties dominated, in different measures, by absorption and scattering (i.e., resulting in different colors in transmission and reflection).^67^ Similarly to the silver-stained glass, also the copper-based red ruby glass was produced through a similar mechanism involving the diffusion of copper ions in the glass and their successive reduction to metallic copper (Figure 1d,e) to form thin red-colored multilayers rich in Cu nanocrystals (Figure 1f), as shown by experimental and archeological evidence.^68,69,76^ All in all, the arcane and highly empirical procedure to form red and yellow colored glass, along with the difficulty to create a highly reducing working environment, ensured that glass fabrication remained exclusive to a limited number of artisans who, though unaware of it, can be rightfully seen as proto-nanochemists.

## Midas’ Touch: Gold

Metallic gold has accompanied the development of human civilization throughout the millennia under different forms. Thanks to its physical properties and its rarity, it has often been imbued with strong symbolism associated with royalty and religion, which partly explains its fate throughout history. For these reasons, this paragraph will be dedicated to exploring the uses of gold, in the form of nanocrystals, in diverse materials and applications.

**Figure 2:**
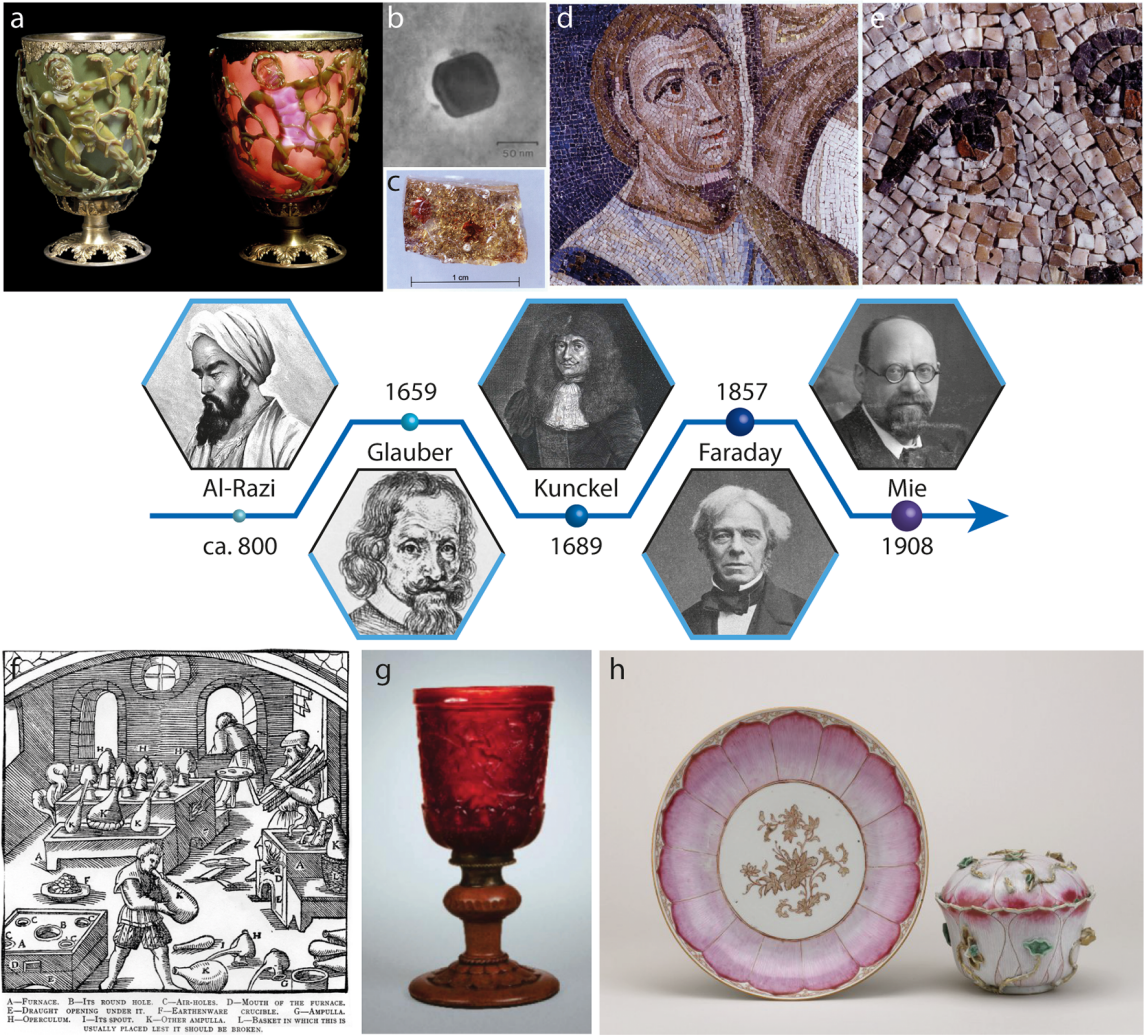
Use of gold nanocrystals throughout the centuries. (a) Photograph of the Lycurgus cup in reflected light (left) and transmitted light (right); reprinted with permission of the British Museum. Copyright trustees of the British Museum. (b) TEM image of a Ag/Au alloy nanocrystal from the Lycurgus cup; reprinted with permission.^81^ Copyright (2007), John Wiley and Sons. (c) Digital image of a Saint Sabina church mosaic *tessera* in Rome, Italy. The gold-foil *tessera* presents few gold ruby droplets from gold nanocrystals precipitation; reprinted with permission.^84^ Copyright Istituto Centrale per il Restauro, photographer Marcello Leotta. (d, e) Face from the Saint Pudentiana church mosaic in Rome, Italy. The flesh tone tesserae are colored by gold nanocrystals dispersed in the glass matrix; reprinted with permission.^84^ Copyright (2010) Susanna Sarmati. Inset portraits (from left to right): Rhazes (al-Razi) (Wellcome Collection. Attribution 4.0 International, CC BY 4.0); Johann Rudolph Glauber (Public domain); Johannes Kunckel (Public domain); Michael Faraday (Public domain); Gustav Mie (reprinted;^87^ Attribution 4.0 International, CC BY 4.0). (f) Representation of the distillation of *aqua valens* from the 1912 Hoover translation of Agricola’s *De Re Metallica* (1556); reprinted with permission.^88^ Copyright (2006) John Wiley and Sons. (g) Photograph of ruby glass goblet (1690–1700) from Potsdam, Germany, probably Gottfried Spiller. CMoG 79.3.258. Gift of The Ruth Bryan Strauss Memorial Foundation. Image licensed by The Corning Museum of Glass, Corning, NY (www.cmog.org) under CC BY-NC-SA 4.0. (h) Digital image of *Famille Rose* porcelain set, Qing dynasty, 18th century; reprinted with permission from the British Museum. Copyright trustees of the British Museum.

Recent studies have investigated the role of gold, in the form of nanocrystals, in the coloration of glass. The earliest report on red-colored glass via the presence of gold nanocrystals is attributed to the Assyrians: Thompson translated a cuneiform clay tablet from 700 BC describing a recipe to make red glass through the addition of gold.^77^ However, it was the Romans who truly mastered the technique of coloring glass in red with gold: the Lycurgus cup, in this sense, represents an exquisite, well-known example (Figure 2a).^78^ The cup depicts a well-known episode from Greek mythology where king Lycurgus, threatening the life of one of the priestesses of the god Dionysus, is restrained by the god himself through the use of vines and is eventually killed. The cup, besides the mythological symbolism, is also interesting because it is a fine example of a dichroic glass: when observed in reflection, it looks green, while it is red when observed in transmission (Figure 2a). The optical properties of the cup have attracted scholarly attention since the second half of the 20th century, when these optical properties had already been correctly attributed to the presence of silver and gold in the glass, possibly in a colloidal form.^79,80^ However, it was only in 1990 that the dichroic properties of the cup were unambiguously attributed to the presence of alloyed Ag/Au metallic nanoparticles in the glass by means of electron microscopy (Figure 2b).^81^ The color is produced by the interaction of light with metallic nanoparticles dispersed in the glass by means of absorption (of the localized surface plasmon modes) and scattering.^82^ Despite the outstanding craftsmanship of this cup, the scarcity of archeological findings of comparable quality let us tend toward the idea that the control of the colorant process was probably very difficult, and similar objects could only be produced in extremely limited numbers.^83^ Nevertheless, the ability to color the glass red via the formation of gold nanoparticles in situ (from which we have the name gold ruby glass or gold red ruby) was preserved during the political and cultural fragmentation of the Roman Empire and was employed to color Roman mosaic tesserae hundreds of years later. A few examples of glass mosaics and panels ranging from the fourth to the 12th century AD have been reported to rely on gold for coloration. In particular, the most typical technique consisted of embedding extremely thin gold foils in glass matrices, thus obtaining a golden appearance. This appearance was enriched and complemented by tiny red droplets produced by the precipitation of colloidal gold in the glass matrix through a thermal process (Figure 2c). However, a more frequent use of gold for coloration is found when analyzing pale, reddish-white colored mosaic tesserae throughout the Mediterranean basin (Figure 2d,e): several studies in fact attribute this color to the presence of colloidal metallic nanoparticles of gold (and mixed alloys of gold–silver) in the glass matrix.^84−86^ The methodology used by the ancient glassmakers to produce these tesserae is still under debate, as is the state of preservation and transmission of this knowledge throughout the centuries, as it is possible that earlier mosaics have been disassembled and their pieces reused for more recent ones. However, it is clear that Roman glassmakers did possess and master the knowledge to produce gold red ruby glass several centuries ago.

More systemic use of gold as a chromophore in glass and ceramics directly emerged from the development of alchemy in the Middle East. This discipline revolved around the search for the philosopher’s stone: according to the tradition, this stone was supposed to transform any common metal into gold, the purest of all metals, and it was often imagined looking like garnets and rubies. In this sense, Ganzenmüller has suggested a link between the search for the philosopher’s stone and the creation of gold ruby glass, where the latter is a product of the former.^89,90^ According to some sparse studies, the ninth century Persian manuscript^91^ “Secret of Secrets”, attributed to the alchemist Al-Razi, contains both one of the earliest modern descriptions of the preparation of gold ruby glass^92^ and one of the oldest recipes for the preparation of pure sulfuric, nitric, and hydrochloric acids, from which *aqua regia* was later prepared.^88,93^ During the 14th and 15th centuries, with the translation and the diffusion of Arabic texts in Europe, the first mentions of gold ruby glass appear in literature, especially in Italy and Germany, even though the truthfulness of these publications is debated.^94^ At the same time, descriptions of how to prepare powerful acids also appear in Europe: in 1530 the alchemist Georg Bauer, better known as Georgius Agricola, described in his *De Re Metallica* how to prepare *aqua valens* (i.e., poweful water, Figure 2f), a generic formulation to indicate powerful acids such as *aqua fortis* (nitric acid) and *aqua regia* (hydrochloric acid/nitric acid, 3:1). The preparation of the latter, capable of dissolving gold, among other things, opened the doors for the preparation of the first dispersions of colloidal gold during the 17th century. In 1685, Andreas Cassius from Leiden published in the *De Auro* a recipe to produce colloidal dispersions of gold nanoparticles from the dissolution of gold in *aqua regia* followed by reprecipitation via the addition of a mixture of stannic and stannous chloride, which took the name “Purple of Cassius”.^94,95^ This preparation has been believed to be the first reported preparation of colloidal gold for a long time; however, Hunt recently showed that Johann Glauber (Figure 2) discovered how to make these preparations at least 25 years before Cassius (in 1659).^96^

The ability to dissolve gold and to produce dispersions of colloidal gold had two major consequences. On one side, strongly fuelled by exoteric and alchemical beliefs, colloidal dispersions of gold have been deployed for medical purposes: diluted solutions of gold dissolved in *aqua regia* (in this context called *Aurum potabile*, potable gold) were believed to be a universal panacea, capable of treating diseases in men and animals.^88,97,98^ On the more technological side, such solution-processing of gold paved the way for the relatively reproducible fabrication of high-quality gold ruby glass. Although some earlier reports exist on producing gold ruby glass from colloidal dispersions of gold (e.g., Antonio Neri), Johann Kunckel (Figure 2) was the first glassmaker able to use this technique to produce gold ruby glass on a large scale (Figure 2g). The son of an alchemist and alchemist himself, familiar with the work of Cassius, Kunckel employed gold chloride, obtained from the dissolution of gold into *aqua regia*, as a precursor. The red ruby glass would then be obtained by reducing the gold chloride in the molten glass via the addition of metallic tin.^64,96^ By varying the thermal annealing steps, he could obtain, very reproducibly, different tints of red. At the end of the 17th century, gold ruby glass became fashionable among the rich elites of Europe, and in the 18th century the production of gold ruby glass expanded in France, with the glass of Bernard Perrot^99^ (a Ligurian glassmaker who used arsenic instead of tin to precipitate the gold), in Italy, with the Murano glassmakers, and in England, where it was produced in a less saturated tint called *cranberry*. The use of gold ruby glass was so widespread that, during the French Revolution, the Convention (the French parliament between 1792 and 1795) considered melting the stained glass from French churches with the purpose of extracting gold, before they discovered that copper was the chromophore, and not gold.^100^ In the meantime, the same solution-based technique for coloring the glass was used to produce gold ruby enamels on ceramics. This craft developed mainly in France, where *La manufacture Royale de Sèvres* gave origin to the *Rose Pompadour* series^101^ in 1757 using Purple of Cassius, and in China, where gold tincture (probably imported by the Jesuits) was used to decorate porcelain, under the name of *Famille Rose* (Figure 2h), from 1735.^94^

During the 18th century the empirical scientific approach introduced by the Enlightenment led to the first investigations of the origin of the color of the Purple of Cassius. In 1857 Michael Faraday (Figure 2) was the first to suggest both that “finely-divided gold” could be at the origin of the red color and that “*a mere variation in the size of its particles gave rise to a variety of resultant colours*”, thus consciously linking, to the best of our knowledge for the first time, color and size in nanoparticles.^102−104^ Despite correctly suggesting the size of the nanoparticles to be smaller than the wavelength of light, Faraday incorrectly attributed the color of the nanocrystals to their relation with vibrations of ether particles: “*Besides, the waves of light are so large compared to the dimensions of the particles of gold which in various conditions can be subjected to a ray, that it seemed probable the particles might come into effective relations to the much smaller vibrations of the ether particles*”. However, it was only in 1898, with the discovery of the slit ultramicroscope by Richard Zsigmondy, that the colloidal nature of gold in the Purple of Cassius was finally unravelled; this discovery earned him the Nobel Prize in chemistry in 1925.^105,106^ To complete the picture, in 1908 Gustav Mie (Figure 2) developed the first rigorous mathematical description of the optical behavior of colloids that scatter light.^107^ According to his theory, when a spherical particle is much smaller than the wavelength of light, an incident electromagnetic radiation can generate a coherent oscillation of the free electron cloud on the surface of the particle, which takes the name of localized surface plasmon resonance. The wavelength of this oscillation, being size-, shape-, and material-dependent, strongly influences the absorption and the scattering of the particles themselves, thus resulting in remarkable optical properties.^14,108−110^

After the abundant use of gold nanocrystals for the coloration of materials over the centuries, the past century has faced the rise of colloidal gold for biological and medical applications. The intrinsic chemical inertia of this metal (i.e., low toxicity), quickly promoted it as an ideal candidate for applications spanning from drug delivery to tumor detection and biosensing.^111−118^ In this case, for example, colloidal gold nanocrystals are widely used to label antigens for biological electron microscopy and for viral antibody and antigen tests (e.g., some Covid-19 rapid tests).^119,120^ The prolificity of this application field is reflected in the rich literature concerning the functionalization of the surface of gold nanocrystals in order to interact with biological probes and receptors.^114,118,121,122^

## Nanocrystals in Ceramics

**Figure 3:**
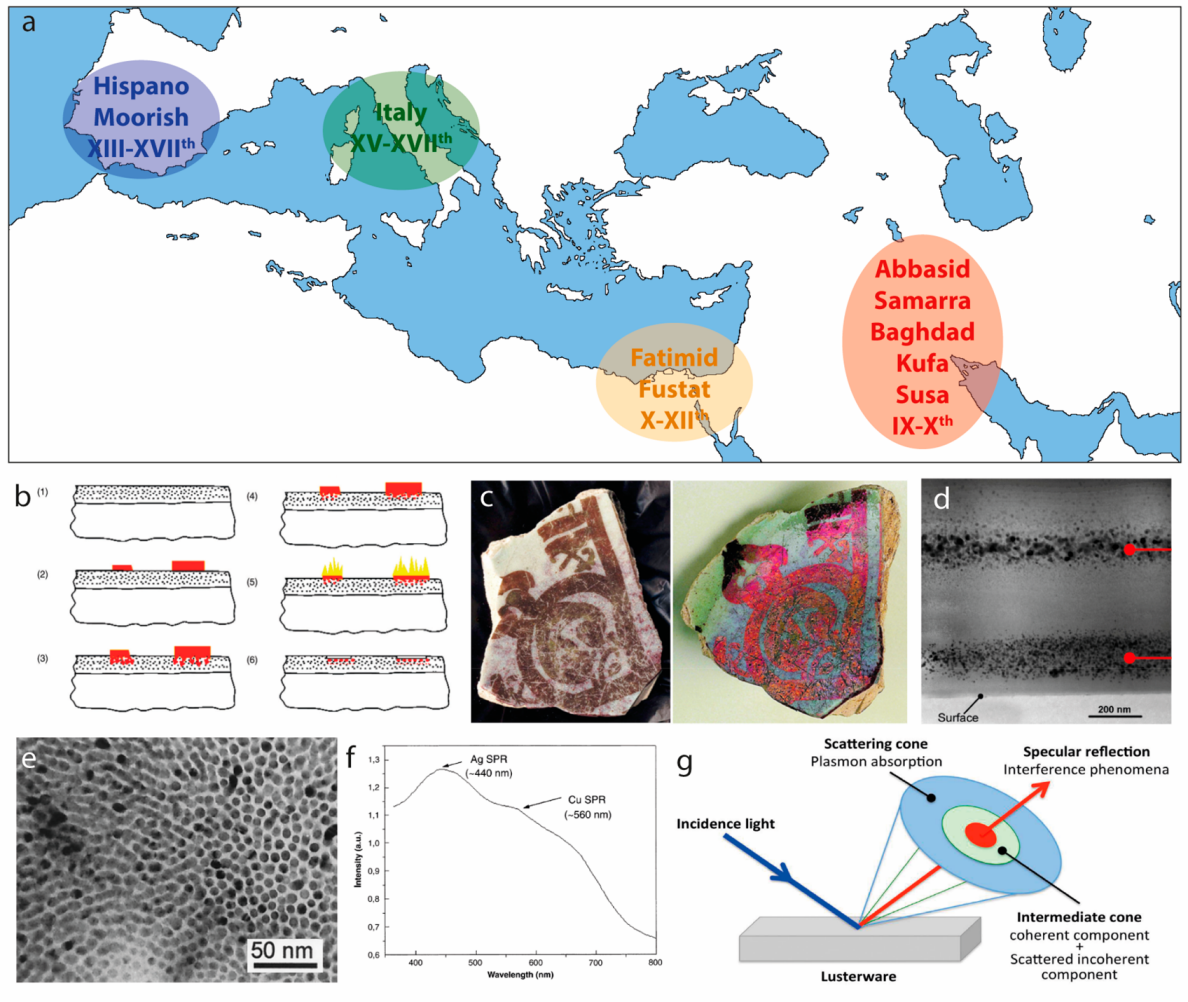
Nanocrystals in luster ceramics. (a) Geographical map showing the location of the main centers of luster ceramic over the centuries. Adapted with permission from ref (128). Copyright (2012) Philippe Sciau. Creative Commons license CC 3.0. (b) Schematic of luster ceramic production: (1) Outermost glassy fired glaze of lead-based ceramic; (2) the precursor paste, containing Ag and Cu salts, lye, vinegar, water is applied onto the glassy surface; (3) the acetic acid, in combination with the lye, attacks the surface, increasing its porosity; (4) the temperature is increased to melt the Ag/Cu-rich liquid; (5) the surface of the pot is locally flashed to burn the residuals of acetic acid and to promote the recrystallization of Ag and Cu as nanocrystals; (6) luster is revealed after washing away the residual paste. Reprinted with permission.^124^ Copyright (2008) with permission from Taylor & Francis. (c) Example of 12th century luster ceramic from Fustat (Cairo, Egypt) at (left) nondiffracting and (right) diffracting observation angles; reprinted with permission.^124^ Copyright (2008) with permission from Taylor & Francis. (d) Transmission electron microscopy image of the multilayered structure of a luster ceramic from the 12th century. Reprinted with permission.^133^ Copyright (2008) Trans Tech Publications, Ltd. (e) Transmission electron microscopy image of the inner part of a 15th century gold-like luster from Deruta, Italy. Reprinted with permission.^134^ Copyright (2004) John Wiley & Sons. (f) Absorption spectrum of a gold-like luster decoration from Gubbio, Italy (16th century). The absorption features associated with localized surface plasmon resonance of Ag and Cu nanocrystals are indicated. Reprinted with permission.^129^ Copyright (2002) Elsevier Science B.V. (g) Schematic representation of the optical effects observed in luster ceramic. Reprinted with permission.^128^ Copyright (2012) Philippe Sciau. Creative Commons license CC 3.0.

The popularity of gold-like decorations on ceramic and glass has been suggested to have fostered the development of luster ceramic. This technique produces a peculiar metallic gold-like glaze on ceramic materials using Ag and Cu; for this reason, this technique might have been developed to circumvent the prohibition, by Islamic rule, of making profane utensils out of gold.^63^ In fact, the first examples of such a luster ceramic or lustreware, characterized by a peculiar metallic glaze, date back to the ninth century AD to the city of Samarra (as well as Baghdad, Basra, Kufa, and Susa), under the domination of the Abbasid Caliphate, in modern Iraq (Figure 3).^123^ After the fracture of the Abbasid Caliphate, luster ceramics disappeared in the Iraqi area to appear in Fustat, Egypt, under the domination of the Fatimids.^73,124^ It has been suggested that, this technique being too difficult to imitate, a direct transmission of knowledge likely occurred.^125^ From this area, luster ceramics spread to Hispano-Moorish Spain, in particular, Valencia,^126^ as did also the manufacturing of paper. In Valencia luster ceramics appeared in the 13th century and survived the centuries of the *Reconquista* until the 18th century, when it was replaced by porcelain from the Orient.^127^ It is probably during this period that the know-how was transmitted to Christian artisans and, from Spain, diffused to Italy in the 15th century, where it became particularly popular in the productions of Gubbio and Deruta.^127−132^

Even though the technique to color ceramics with nanocrystals is in many ways similar to that used to produce colored glass, the resulting structure and optical properties are quite different due to the nature of the material. A very scarce number of recipes have survived the passage of time. Nevertheless, via the investigation of surviving samples and via experimental archeology,^135−138^ we now have an understanding of how luster ceramic was produced.^124^ Typically, a paste of clay and ochre, Cu and Ag salts, water, vinegar, and lye (likely with an excess of vinegar) was applied to the outer vitrified surface of the ceramic pot (Figure 3b). During this step, the acetic acid of the mixture would partially dissolve, at 80–100 °C, the Pb-rich surface of the pot, effectively increasing its porosity and therefore maximizing the diffusion of the Ag/Cu-rich melt. This step would be followed by an increase in the temperature to allow the silver and copper salts to diffuse into the glass matrix, where they would undergo an ionic exchange with the alkaline ions (K^+^ and Na^+^) composing the ceramic. This process would produce layers of metallic Ag and Cu nanocrystals of different shapes and sizes.^123,139,140^ To promote the reduction to metallic silver and copper, iron and tin oxides would be added to the ceramic paste. Several “firing” steps at different temperatures (600–1000 °C) would then follow in order to burn the acetic acid residuals and melt the Ag and Cu, allowing them to reprecipitate as nanocrystals so to obtain the targeted coloration; then the remaining paste would be washed away from the surface, thus leaving a beautiful iridescent color (Figure 3c).^125^ This technique, compared to that used to color glasses, would result in several nanometers-thick multilayers (spacing of few hundreds of nm)^133,134^ of highly concentrated nanocrystals (Figure 3d,e).^124^ For this reason luster ceramic has been viewed by many as the first example of a high-density nanocluster thin film ever developed by humans.^134^ Furthermore, several reports^141−144^ state that the multilayered structure, combined with the presence of silver and copper nanocrystals, is the origin of the angle-dependent coloration of luster ceramics. In particular, while the absorption of the localized surface plasmon resonances of the nanocrystals (Figure 3f) produces a diffused incoherent coloration, the interference of the multilayered structures generates a strong angle-dependent coloration that is tuned by the interlayer distance (Figure 3g).

Historical samples of luster ceramics are very heterogeneous both geographically and chronologically: while the early productions of Egypt and Mesopotamia usually have a more complex structure along with smaller nanocrystals (10–15 nm), the later samples from Spain and Italy have simpler structures with larger nanocrystals (50–100 nm).^128^ This difference is a result of the fact that, although in all the productions, Ag and Cu (and Ag/Cu alloys) are the chromophores (typically Ag for yellow color and Cu for red), different manufacturing techniques were used. This is mainly demonstrated by the presence of different elements and impurities in the glaze itself. Typical of the Italian production is, for example, the presence of Bi to reduce silver and copper,^127^ while the presence of FeO and HgS impurities has been connected to the balancing of the chemical environment between reducing and oxidizing.^137^ Tin and lead would be further added to the glass to tune the diffusion of the ions and the reducing power of the glass, thus resulting, when combined with different firing steps, in nanocrystals of different sizes.^145,146^ As a result, different glass compositions and firing techniques would result in different colors for the same amount of chromophores added.^141^

Overall, the production of luster ceramics required a high level of technical skill as well as strong empirical knowledge from craftsmen of the Middle Age. These proto-solid-state chemists had to subtly balance all these experimental parameters in order to produce high-quality ceramics in a process that, in the words of a 16th century potter, “is so uncertain that often, out of one hundred pieces, hardly six are good”.^147^

## Beauty and Health

Care of the body has been one of the main focuses of mankind throughout the whole of human history. It is, therefore, natural to find examples of the use of nanocrystals for healthcare purposes. In the previous paragraphs, we have already discussed the use of colloidal dispersions of gold during the Modern Age as a panacea; however, supposed healing properties were not a prerogative of gold, and other metals and compounds have been used for similar purposes.

It is well-known that galena, the natural mineral form of PbS, was widely used in the ancient world as a pigment.^148^ It therefore does not come as a surprise that PbS is also one of the earliest pieces of historical evidence for the use of semiconductor nanocrystals. In particular, it has been shown that, during Greco-Roman times, a mixture of PbO, Ca(OH)_2_, and water was used as a hair dyeing mixture.^149^ Researchers showed that the alkaline environment created by Ca(OH)_2_ in water would promote the reaction of PbO with the sulfur-based amino acids of keratine, thus producing in situ nanocrystals of PbS. The nanocrystals, with an average size of ∼5 nm, would blacken the hair while retaining its mechanical properties. Another popular use of galena was as a component to make *kohl*, a powder cosmetic comparable to modern mascara widely used by the Egyptians.^150^ Although there is no scientific evidence to support the statement that PbS was used for this purpose in its nanocrystalline form, we do not think this theory is to be rejected completely. We note that, until the modern age, there was no awareness about lead toxicity; this explains the widespread use of lead for various applications, ranging from plumbing to artificial sweeteners.^151^ A cosmetic for the eyes similar to *kohl*, *kajal*, is widespread in India for its supposed antifungal and antibacterial properties. *Kajal* is prepared from the incineration of the Monosha plant leaves covered in oil, and it has been shown to contain carbon nanocrystals with sizes below 100 nm.^150,152^ More generally, a category of preparations of the traditional Indian Ayurvedic medicine, the *Bhasmas*, make much use of nanocrystals for therapeutic purposes.^47^*Bhasmas* are described as herbo-mineral preparations (i.e., consisting of grinded metals and herbs) produced through incineration, and they are employed to treat several diseases.^153^ The metals used for the *Bhasmas* are really diverse (e.g., Zn, Pb, Hg, and, obviously, Au), but they have been shown to enhance the absorption by the human body (i.e., assimilation) of the active ingredients contained in the herbs thanks to their nanoscale dimensions.^154−156^

Interestingly, the use of metals for therapeutic and disease-preventative reasons is not limited to the Indian subcontinent. Several literary sources show how the antimicrobial properties of silver were already well-known from the sixth century BC, when water was often stored in silver containers;^157^ for the same reason, silver nitrate was prescribed by medieval doctors for the treatment of wounds.^158,159^ Despite this widespread knowledge it is striking that there is no evidence for the use of silver in its colloidal form in history, unlike colloidal gold. The few mentions that exist are not supported by factual evidence.^45,160^ The well-known antibacterial properties of silver nanoparticles has recently promoted them as a common ingredient in modern deodorants and toothpastes, along with gold nanoparticles.^161^ However, the current use of nanocrystals in cosmetics is not only limited to silver and gold: at present a wide variety of nanocrystals are widely used in this industry.^162^ We have already mentioned the use of various nanocrystals (e.g., TiO_2_, ZnO, CeO_2_, and ZrO_2_) in sunscreens due to their strong light absorption in the UV region, but for the same reasons they are also extensively used in lip balms and moisturizers.^163^ Furthermore, several modern cosmetic products rely on nanocrystals (e.g., in the form of nanocapsules or nanosuspensions) as agents to carry and manipulate the release and action of the cosmetic active substances.^162,164^ In particular, nanocrystals offer superior properties compared to bulk materials, for instance, improved penetration in the skin and in the mucosal surfaces, as observed for organic nanocrystals such as Rutin (i.e., quercetin-3-*O*-rutinoside) nanocrystals.^165^

## Semiconductor Nanocrystals in the Modern Age

The early 1980s mark the inception of research in the field of semiconductor nanocrystals (or quantum dots).^166^ Indeed, four decades ago, the size-dependent optical properties of solvent- and glass-dispersed colloidal semiconductors have been unambiguously attributed to the quantum-size effect. Nevertheless, it is also instructive to highlight the earlier history and the continuity of research that led to the modern field of quantum dots.

**Figure 4:**
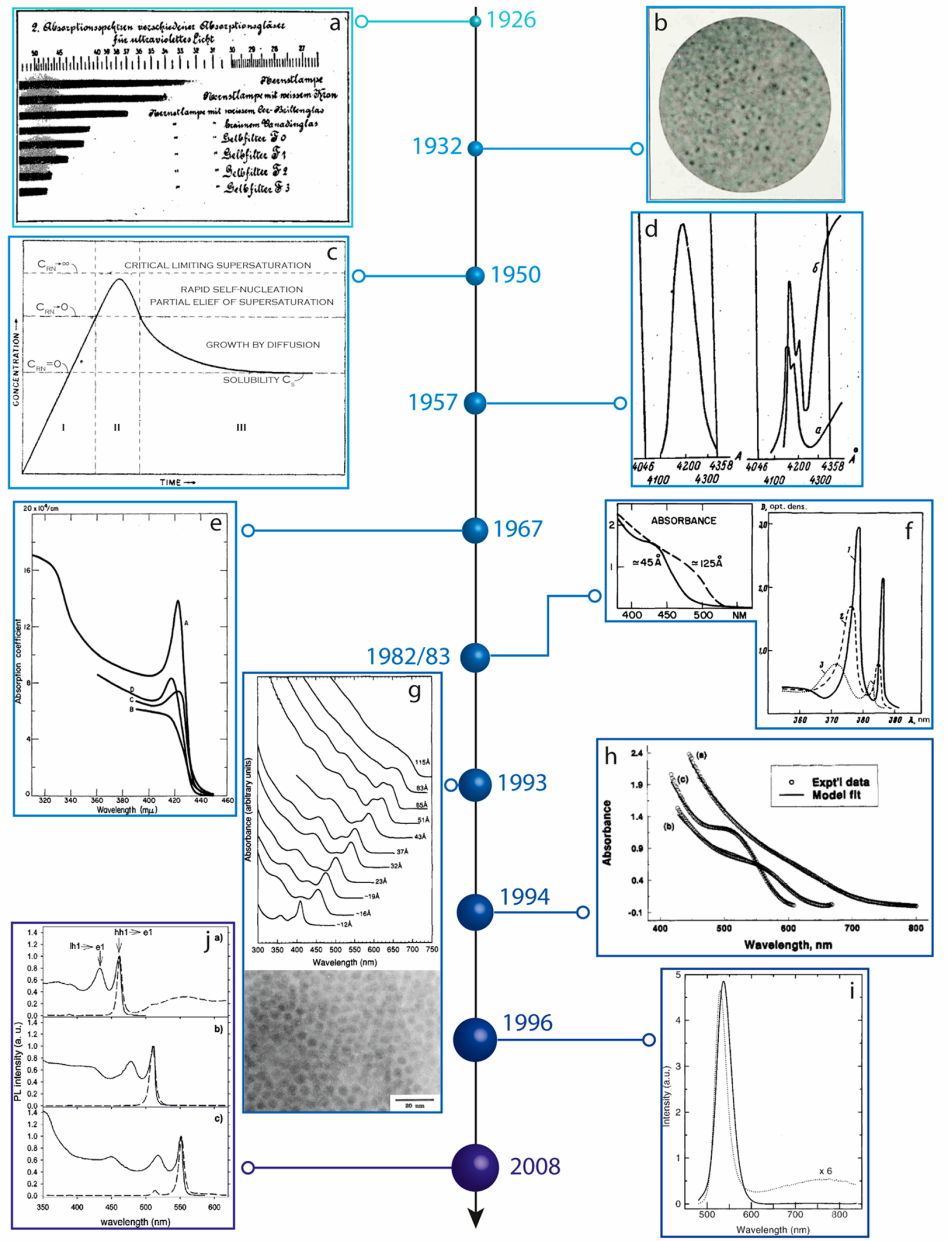
Semiconductor nanocrystals in the Modern Age. (a) Absorption spectra of different glasses containing CdS. Reprinted with permission.^167^ (b) Micrographs of selenium-containing ruby-colored glasses (magnification × 5000). The black dots are CdSe microcrystals in the glass matrix (smallest size ∼250 nm). Reprinted with permission.^168^ Copyright (1932) Society of Glass Technology. (c) Schematic representation of the concentration of monomers as a function of time during the synthesis of hydrosols; adapted with permission.^169^ Copyright (1950) American Chemical Society. (d) Emission spectra of CuBr/NaBr mixtures showing narrow line width emission, associated with the presence of quantum dots. Reproduced with permission.^170^ Copyright (1957) AIP Publishing. (e) Absorption spectra of AgI (nano)crystals of different sizes. The sharp absorption features are associated with excitonic features. Reprinted with permission.^171^ Copyright (1967) American Physical Society. (f) (left) Absorption spectra of CuCl nanocrystals, with a radius between 2.5 and 31 nm, embedded in a glass matrix. The sharp excitonic features become more prominent with smaller sizes. Reprinted with permission.^172^ Copyright (1983) AIP Publishing. (right) Absorption spectra of CdS nanocrystals dispersed in water. The size dependence of the absorption edge is clearly visible. Reprinted with permission.^173^ Copyright (1982) JETP Letters. (g) (top) Absorption spectra of colloidal CdSe nanocrystals ranging from 1.2 to 11.5 nm. (bottom) Transmission electron micrograph of colloidal nanocrystals. Reprinted with permission.^174^ Copyright (1993) American Chemical Society. (h) Absorption spectra of InP nanocrystals. Reprinted with permission.^175^ Copyright (1994) American Chemical Society. (i) Photoluminescence of CdSe (dotted line) and CdSe/CdS (solid line) nanocrystals. Reprinted with permission.^176^ Copyright (1996) American Chemical Society. (j) Absorption (solid lines) and photoluminescent excitation (dotted lines) spectra of nanoplatelets of different thicknesses. Reprinted with permission.^177^ Copyright (2008) American Chemical Society.

During the 19th and 20th centuries, interest in glass manufacturing received a substantial boost; the development of optical microscopy and spectroscopy, in fact, promoted research oriented to the production of high-quality glass, both colored and transparent. For example, at the end of the 19th century, Otto Schott established the “Glastechnisches Laboratorium Schott & Gen Jena”, which soon became the leader in optical glass manufacturing, to the point that “Schott glasses” soon, by the transmission of meaning, became the term to refer more generally to optical filters.^178,179^ In this context, as the heirs of a multimillennial tradition from artists and craftsmen, researchers were investigating the composition dependence (via the incorporation of additives such as chalcogens and halogens) on the optical properties of glasses. To the best of our knowledge, it is in this framework that researchers in the field of glass coloration started to observe size-dependent optical properties in glasses containing CdS additives (Figure 4a).^167^ In particular, they noticed that the absorption edge and emission of the colored glasses could be tuned by temporally varying the tempering step. As the works of Zsigmondy and Mie were already known, the authors hypothesized that the origin of the color shift toward longer wavelengths was to be found in the formation and growth in size of CdS colloids in the glass, producing, to the best of our knowledge, the first observation of quantum confinement effects in semiconductor nanocrystals. A similar observation was made a few years later for samples of ruby-colored Se-containing glass.^168^ In this case, researchers correctly attributed the coloration of the glass to the precipitation of CdSe and CdS crystallites in the glass itself and recorded one of the first micrographs of sub-micrometer semiconductor crystals (Figure 4b). Moreover, they hypothesized a link between the color variation (from pale yellow to red) observed during tempering of the glasses and a variation in the size of the crystals over time.

In 1950, Victor LaMer developed a theory explaining the formation of monodisperse hydrosols through a separation between nucleation and growth (Figure 4c). This theory, as we will see, has been pivotal in the development of monodisperse colloidal nanocrystals.^169^ In the second half of the 20th century researchers started focusing on the investigation of the size-dependent optical properties of (nano)crystals embedded in solid matrixes. In a few years crystals of very different compositions, and in matrices or as colloids, were characterized: CuBr (Figure 4d),^170,180^ CuCl,^181^ AgI, and AgBr (Figure 4e).^171,182,183^ For CdSe nanocrystals (with radius between 1 and 5 nm) dispersed in glass, the inverse size dependence of optical absorption with the squared radius of the nanocrystals was classified as an “optical anomaly” by Katzschmann.^184^ Similar observations on size-dependent optical properties were also collected in the case of thin films; for example, Stasenko unambiguously identified the dependence of the energy band gap of CdS on the thickness of the thin films (1.7 nm).^185^ In 1984, Itoh and co-workers observed the effects of exciton confinement in CuCl microcrystals embedded in NaCl matrixes.^186^ All of these observations throughout the decades, together, paved the way for the work of Alexey Ekimov and Louis Brus, who successfully and intentionally synthesized semiconductor nanocrystals in glass and in water and correctly attributed their size-dependent properties to the quantum confinement effect (Figure 4f).^172,173,187−190^ Furthermore, collaborations between Ekimov and Alexander Efros resulted in the very first theory describing the “quantum size effect” in semiconductor nanocrystals.^191,192^ The size-dependent optical properties were linked to the concept of the confinement of the exciton wave function into a potential well smaller than the exciton Bohr radius. Interestingly, despite independently investigating and discovering quantum dots, these two groups of scientists (Ekimov’s/Efros and Brus’) approached the quantum dot field as successors of two different research currents. Ekimov was investigating the physicochemical properties of halogen-doped colored glasses, which, as we previously showed, derives from a long-standing tradition of colored (optical) glass manufacturing. Brus’ research instead was rather motivated by the anticipated utility of colloidal semiconductors in photocatalysis.^193−199^ In both cases, it is essential to highlight the role of the cross-fertilization of ideas across scientific disciplines (i.e., the influence of concepts present in a specific scientific discipline in the development of a new idea in a different scientific discipline). In particular, Brus was also influenced by the contemporary observations of the optical effects in molecular-beam-epitaxy-grown quantum wells (one-dimensional (1D) quantum confinement).^179,200^ The independent research of these two groups of scientists (Brus’ and Ekimov’s/Efros) represents a milestone for the quantum dot field: their work correctly linked, for the first time, empirical evidence with scientific understanding of the underlying process. Furthermore, their work was crucial in sparking the interest of the scientific community and successfully igniting research in this field. This milestone has been recently described in a highly detailed review by the leading actors themselves;^179^ we refer to this work for a more specific and more personal account of their discoveries.

In the years immediately after Ekimov’s and Brus’ publications, the field saw a rapid acceleration: the colloidal synthesis was extended to a plethora of other semiconductors,^201−203^ while the nanocrystals began to be extensively characterized both optically and structurally.^204,205^ However, the main bottleneck to the complete establishment of the field was the high polydispersity of the nanocrystals produced, which hampered efforts to consistently link the optical properties to a well-defined size of the nanocrystals. This problem was effectively solved in 1993 for NCs both in glass and in solution. Ekimov solved this impasse for NCs in glass by using different stages, characterized by different temperatures, in the production of the nanocrystal-doped glass, thus separating the nucleation and growth phases of the nanocrystals; this technique decreased the size dispersion from 15% to 5%.^206^ A similar approach was independently developed for colloidal quantum dots. Inspired by the theory of LaMer, the research group of Bawendi developed the highly reliable synthetic method known as “hot injection” for the production of monodisperse colloidal nanocrystals in high concentrations (Figure 4g).^174^ This work represents another key benchmark in the development of this field, since it allowed the production of consistently monodisperse (semiconductor, but also metal and oxide) nanocrystals with different chemical compositions.

The year after, in 1994, the first synthetic procedure to produce InP colloidal nanocrystals was published (Figure 4h), thus introducing what would become one of the main competitors to the II–VI semiconductor NCs family.^175^ On the path toward better technical developments, in 1996 the very first core/shell nanocrystals were synthesized. This enabled the possibility to tune the quantum confinement effect by varying the shell thickness and material, opening the doors to core/shell heterostructures with spatial delocalization of the charges. Furthermore, the possibility to confine the exciton in the core of these heterostructures (i.e., away from surface defects) permitted one to achieve much higher quantum yields and, overall, to make nanocrystals more chemically stable (Figure 4i).^176^

The next important milestone was the transition, in the years 2000–2010, toward safer and more benign precursors. Organometallic reagents were thus substituted with carboxylates, while, for example, elemental selenium was introduced as a potent selenium source.^207−213^ The possibility to produce high-quality quantum dots with commonplace chemical equipment (e.g., with Schlenk lines, fume hoods) and safe reagents made them readily accessible to researchers of different disciplines, promoting the interdisciplinarity of the quantum dot field. From this point onward, the number of publications grew exponentially, mainly thanks to interdisciplinary collaborations between synthetic chemists, physicists, and engineers.

Among significant subsequent advances, we mention the development of anisotropic particles: colloidal nanorods in 2000 by Peng^214^ and colloidal semiconductor nanoplatelets by Ithurria in 2008 (Figure 4j).^177,215^ These types of nanocrystals, characterized by atomically defined thickness (for nanoplatelets) and strong exciton confinement in only one or two directions, are particularly important because they introduced the shape of the nanocrystals as an additional parameter toward the manipulation of the optical properties of semiconductor nanocrystals (in addition to their size).^216^ Furthermore, from the mid-2000s researchers also started to work on the development of organic^217−219^ and inorganic^220^ ligands to deploy semiconductor nanocrystals in commercial applications. Thanks to these new ligands, charge transport in quantum dot solids was strongly improved, and quantum dots could finally be efficiently used for optoelectronic applications involving the production and harvesting of light. It has to be noted, though, that the long-term stability of quantum dots is still a major issue, hampering their effective widespread use for commercial applications. Notable progress in this field is seen as the very first quantum dot displays in the past decade.^19^ The continued emergence of size- and shape-uniform nanocrystals led to their use as “artificial atoms” for constructing mesoscale crystalline structures by the spontaneous self-assembly of nanocrystals. These superlattices are of mono, binary, or ternary compositions and exhibit periodic and quasi-crystalline order.^221−225^ Such efforts pave the path to tailoring collective physical properties emerging from the close packing of nanocrystals and their periodic arrangements, such as miniband transport and super-radiance.^29,226−229^

Lead-halide perovskite (LHP) semiconductor nanocrystals are the most recent generation of colloidal semiconductors.^230,231^ In particular, their near-unity photoluminescence quantum yield without any shell passivation,^232^ their intriguing optical properties on the single-particle level^233,234^ as well as on a collective scale,^226^ and a facile synthesis quickly promoted them to become one of the most interesting materials for scientists in the field of semiconductor nanocrystals.^235^ As we have tried to show with this review, the evolution of the scientific knowledge in the field of nanocrystals can be traced back to much earlier times, as the product of a continuity of observations and discoveries; this is also true for perovskite nanocrystals.

**Figure 5:**
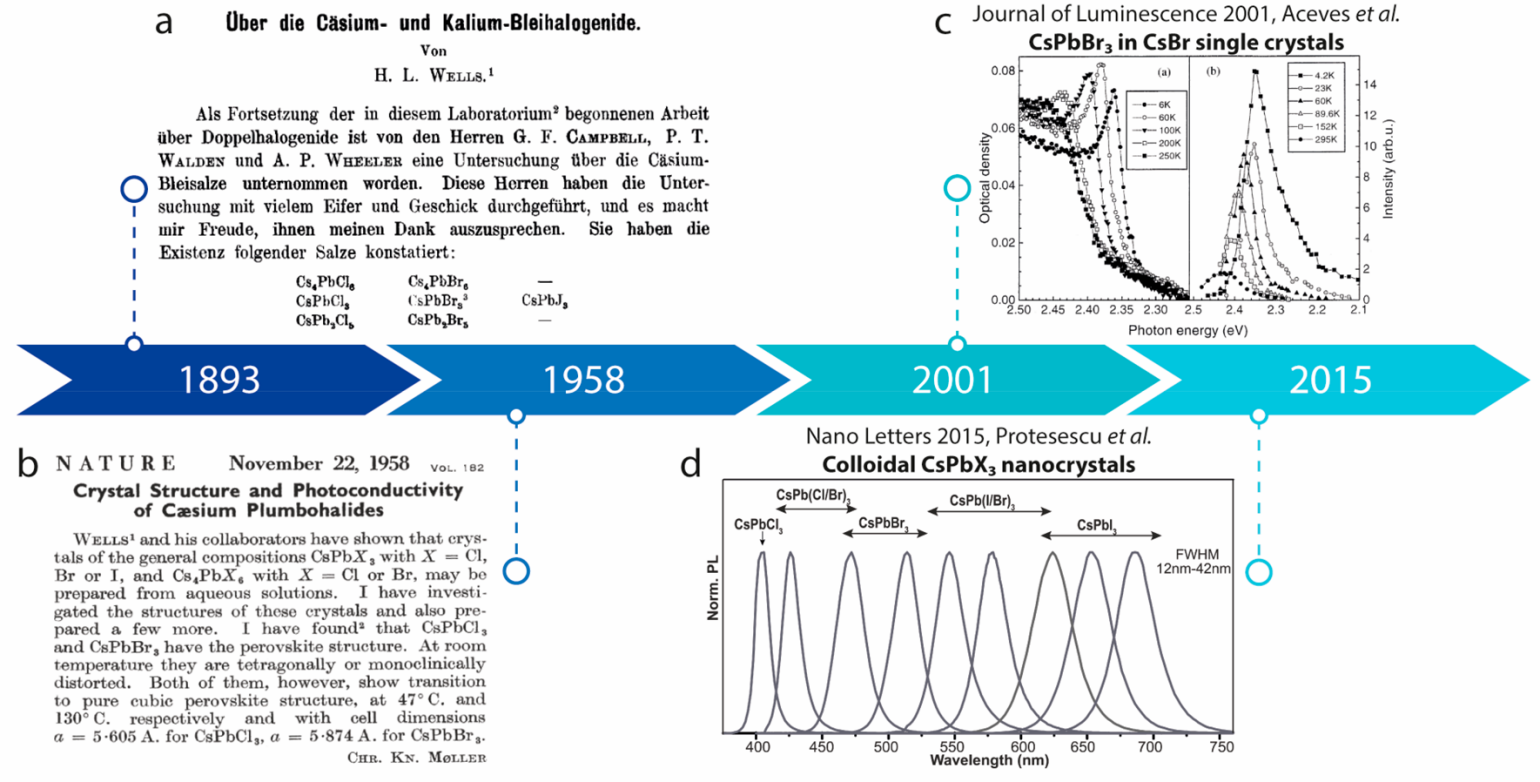
Perovskite nanocrystals. (a) Header of the scientific article by H. L. Wells, where cesium-based LHPs are mentioned for the first time. Reproduced with permission.^236^ Copyright (1893) Verlag GmbH & Co. KGaA, Weinheim; with permission from John Wiley and Sons. (b) Header of the scientific article by Møller in 1958, when the crystal structure of CsPbX_3_ was identified for the first time as perovskite. Reproduce with permission.^237^ Copyright (1958) Nature Publishing Group. (c) Absorption (left) and photoluminescence (right) of CsBr/Pb 0.05 mol % samples measured at various temperatures. Reprinted with permission.^238^ Copyright (2001) Elsevier Science B.V. (d) Photoluminescence of CsPbX_3_ colloidal nanocrystals. Adapted with permission.^232^ Copyright (2015) American Chemical Society.

The first mention of cesium-based lead halide compounds is attributed to H. L. Wells, who identified them and prepared them from aqueous solutions in 1893 (Figure 5a).^236^

However, it was only at the end of the 1950s that the crystal structure of bulk CsPbX_3_ (X = Cl, Br, I) was clearly identified as perovskite. At the same time, the first observations of a crystalline phase transition and photoconductivity properties were recorded (Figure 5b).^237,239−241^ Relevant for the development of colloidal cesium-based perovskite nanocrystals was the research investigating the optical properties of bulk cesium halide doped with lead. At the latest in 1976,^242^ investigators observed that any excitation in the absorption band of cesium bromide, doped with lead, at room temperature resulted in one single emission band at 2.45 eV (green region). This photoluminescence is now attributed to the inclusion of CsPbBr_3_ nanocrystals in the CsBr matrix. In the 1990s, in fact, the concept of the confinement of the excitons was invoked to explain the optical properties of lead halide perovskites, and spectroscopical measurements were performed on few-nanometer large CsPbX_3_ inclusions in CsCl, CsBr, and CsI hosts.^238,243−249^ For the first time, the concept of “quantum size effect” was introduced to explain the discrepancy between the optical properties of these inclusions and the corresponding CsPbX_3_ bulk (Figure 5c).^238^ In general, starting from 1997, the bright emission of CsPbBr_3_ nanocrystals, formed in situ in CsBr/Pb matrixes, was investigated systematically both in thin films and single crystals.^238,244,246,250−253^ At the same time, also thin films of stoichiometric CsPbX_3_ were studied.^254−257^ The interest for thin films was also boosted from the fact that this geometry, combined with the high density of emission centers, was ideal for stimulated emission experiments (lasing).^258−260^

Analogously to conventional quantum dots, also in the case of cesium-based perovskite nanocrystals the conceptualization of quantum dots in solid matrixes was the prelude to the development of colloidal nanocrystals. Inspired by the aforementioned works as well as by the surge in attention for methylammonium lead halide compounds for photovoltaic applications,^261−263^ in 2015, size-tunable, monodisperse, and shape-uniform CsPbX_3_ nanocrystals were produced for the first time (Figure 5d).^232^ The extraordinary control on their photoluminescence, their near-unity quantum yield, and their facile synthesis quickly captured the interest of researchers, thus promoting them as the herald of a new generation of colloidal quantum dots. Soon after, the colloidal synthesis was extended to other lead-based perovskite materials, such as formamidinium-^264^ and methylammonium-^265^ based LHP, whose bulk forms were already known from much earlier.^266−268^

The first commercial application of semiconductor nanocrystals appeared in 2013, when Sony developed the first display employing quantum dots (from QD Vision–CdSe-based) to improve the light-emitting diode (LED) backlighting in LCD televisions so to achieve a better color gamut.^19,269^ Since then, quantum dot-based displays have slowly penetrated the market, and they can now be found in upmarket displays distributed mainly by Samsung (who acquired QD Vision in 2016) under the “QLED” brand (using InP-based quantum dots instead of the CdSe-based ones).^270,271^ In the foreseeable future novel quantum dot displays are expected to penetrate the market, where quantum dots are employed as color filters/converters; in this light should be seen the integration of QLED and organic light-emitting diode (OLED) technologies, as announced by Samsung,^272^ and the use of LHP nanocrystals, as developed by several companies (e.g., Avantama, Nanolumi, Helio Display Materials, PeroLED, BrightComSol, Quantum Solutions). Furthermore, if the main issues related to stability over time can be overcome, quantum dots can also be expected to play a pivotal role in the development of micro-LEDs,^273,274^ flexible displays,^275^ and displays based on an electroluminescent quantum dot film.^276^ Notably also infrared-emitting quantum dots have recently found a commercial deployment; in particular, Quantum Solutions, and other companies, currently sell them (i.e., PbS nanocrystals) as colloidal materials, and Emberion has introduced them in infrared cameras and other solid-state devices.

## Conclusions

Currently, nanocrystal research proceeds at a vertiginous speed, as witnessed by the increasing number of scientific publications published each year, delivering new findings and opening up new questions at a pace never before experienced in the history of mankind. We are now able to produce nanocrystals, both in solid matrices and in solution, with exquisite size and morphological control and diverse properties, allowing us to manufacture objects that would have been attributed to magic by our ancestors.^277^ However, in our progress, we must not lose the retrospective view of the path that led us here. The history of nanocrystals is not just defined by a few scarce breakthroughs but is also the product of a continuous process of improvement and refinement that, from the technical crafts of our ancestors, leads to modern-age laboratories.

We view this interplay between past and present as crucial for informing and inspiring novel research that is also grounded on insights from the past. In particular, we envisage three main directions along which future research in this vein should develop: (1) investigating the link between the nature of nanocrystals (i.e., size and composition) and their optical properties in ancient artifacts, (2) retrieving and reproducing ancient recipes and methods for the production of such artifacts, and (3) tracing the evolution of knowledge through the centuries by reviewing historical scientific accounts through the lens of our modern theoretical understanding. We believe that this can only be achieved through a combined interdisciplinary effort from chemists, physicists, materials scientists, and historians, in order to unveil the legacy that, through the millennia, connects us to our ancestors.

## Vocabulary

Nanocrystal: a piece of material whose dimensions (at least one) are in the nanometer (10^–9^ m) range and whose atoms are arranged in an ordered structure
gold ruby glass: a red glass whose coloration originates from the presence of gold nanocrystals embedded in the material
localized surface plasmon resonance: a coherent oscillation of the free electron cloud on the surface of a nanoparticle, induced by light excitation, and confined by the size of the nanoparticle being smaller than the wavelength of the light used to excite the plasmon
luster ceramic: a particular type of ceramic and pottery characterized by a gold-like glaze and produced via the incorporation of silver and copper in the material
exciton: a bound state of an electron and a hole, also called electron–hole pair, attracting each other via electrostatic Coulomb interaction
quantum confinement: a variation of the properties of a material due to the confinement of the exciton into a piece of material whose dimensions are smaller than the exciton Bohr radius
